# Effectiveness of Multisensory Stimulation on Cognitive Function in Older Adults With Mild Cognitive Impairment: Protocol for a Systematic Review

**DOI:** 10.2196/88720

**Published:** 2026-05-14

**Authors:** Lin Qiu, Xiaoyi Wang, Wen Gao, Zhonglin Zhou, Wenjuan Ji, Haoxu Zuo, Tianhao Xu, Shuo Liu

**Affiliations:** 1 Department of Nursing, School of Public Health and Nursing, Hangzhou Normal University, No.2318 Yuhangtang road, Yuhang District, Hangzhou, Zhejiang, 311121, China, 86-571-28865322; 2 Department of Nursing, First Affiliated Hospital Zhejiang University, Hangzhou, Zhejiang, China; 3 School of Clinical Medicine, Hangzhou Normal University, Hangzhou, Zhejiang, China

**Keywords:** multisensory stimulation, mild cognitive impairment, older adults, cognitive function, protocol, systematic review

## Abstract

**Background:**

Mild cognitive impairment (MCI) is an intermediate state between normal cognitive aging and dementia, with a global geriatric prevalence of 23.7%. Multisensory stimulation (MSS) has emerged as a promising approach to improve cognitive impairment. However, relevant research is heterogeneous, and the evidence has not yet been systematically synthesized.

**Objective:**

This study aims to systematically review the evidence on the effectiveness of MSS on cognitive function in older adults with MCI, appraise different MSS interventions, identify limitations in study design and implementation, and provide recommendations for future trials.

**Methods:**

A comprehensive search will be conducted in PubMed, Embase, PsycInfo, Web of Science Core Collection, and the Cochrane Library, with manual screening of reference lists and gray literature. Eligible studies are randomized controlled trials that involve adults aged 60 years or older with confirmed MCI and evaluate intentional MSS with reported parameters. Two reviewers will independently select the studies, extract the data, and assess risk of bias using version 2 of the Cochrane risk-of-bias tool for randomized trials. The primary outcomes are global cognitive function, memory, executive function, and attention; secondary outcomes include quality of life, adherence, and safety. A meta-analysis will be performed using mean differences or standardized mean differences if the studies are sufficiently comparable in population, interventions, and outcomes; otherwise, a narrative synthesis will be conducted.

**Results:**

This protocol has been registered in PROSPERO, and the methodological frameworks were finalized on October 5, 2025. The systematic literature search was completed between November 1, 2025, and December 31, 2025, followed by study screening and data extraction between January 1, 2026, and February 28, 2026. Data synthesis and final manuscript preparation are expected to be completed by April 30, 2026.

**Conclusions:**

The systematic review is expected to provide a comprehensive synthesis of the evidence on MSS for older adults with MCI and will help identify the optimal intervention parameters and implementation strategies. The findings may help inform future trial design and the optimization of nonpharmacological interventions for cognitive health in older adults with MCI.

## Introduction

### Background

Mild cognitive impairment (MCI) is a clinical intermediate state between normal cognitive aging and dementia, characterized by a decline in one or more cognitive domains. With the global increase in the aging population, the burden of MCI continues to increase. The global prevalence of MCI in the geriatric population has been reported to be 23.7% [1]. Notably, MCI imposes a substantial economic and social burden at the “predementia” stage. Compared to healthy adults, patients with MCI have an average 85% higher cost from a payer’s perspective in addition to a 170% increase in societal costs, which are mainly driven by formal and informal care [2]. However, not all patients with MCI develop dementia. Cohort studies have indicated that a subset of older adults with MCI can transition to normal cognitive function, whereas others remain stable without dementia progression [3,4]. Considering the substantial burden of MCI and its potential for reversion to normal cognition, developing and implementing targeted interventions to delay MCI progression and promote cognitive recovery in older adults is vital.

Currently, nonpharmacological interventions (NPIs) such as physical exercise and cognition-based and combined multimodal interventions serve as the mainstream strategies for cognitive intervention in MCI [5,6]. Among these, digital cognitive training (DCT) has emerged as a potential and safe option. A meta-analysis found that mobile device–based electronic games were effective in slowing down the decline in global cognition and executive function [7]. A network meta-analysis of 14 randomized controlled trials (RCTs) reported that serious digital games were more effective than traditional training methods in improving cognitive ability and daily behavioral capacity [8]. Virtual reality (VR) has also exhibited positive effects on crucial cognitive domains such as attention, executive function, and global cognition [9].

Despite the increasing evidence supporting the effectiveness of DCT for MCI, 2 critical challenges persist. First, a lack of rigorous, cost-effective intervention protocols and clinical guidelines that incorporate evidence-based recommendations for the design and implementation of DCT has been observed [10], and practical adherence exacerbates this gap. A recent study found that most participants failed to complete the standard dose of DCT because of a disparity between their expectations and reality [11]. Second, although most digital interventions share similar core training content (eg, memory and executive function tasks), their effectiveness varies significantly [12].

The main reason for this variation is the ambiguous underlying mechanism of DCT, which is compounded by its reliance on sensory presentation. Although a dose-response relationship exists between the delivery mode and effectiveness of cognitive training [13], existing studies rarely address how the sensory input format influences outcomes. This oversight is critical because multisensory integration is compromised in cognitively impaired populations [14]. Compared with adults experiencing healthy aging, older adults with MCI are more often auditory dominant, and multisensory gains are related to MCI performance [15]. Considering this, the systematic synthesis of multisensory stimulation (MSS) modes in existing studies may help identify effective sensory combinations, thereby helping establish a framework to guide the design of effective DCT protocols for MCI.

Furthermore, MSS refers to the interventions that simultaneously activate 2 or more visual, auditory, olfactory, tactile, and other sensory channels [16,17]. Effective cognitive performance (eg, navigation and social interaction) relies on the brain’s real-time dynamic integration and processing of multisource information, such as visual, auditory, and proprioceptive inputs [18]. From a neurobiological perspective, existing interventions may partially activate specific brain networks and fail to fully activate the neural channels necessary for sustained cognitive improvement [19]. Consequently, clarifying the benefits for distinct cognitive domains and long-term intervention sustainability is necessary [20].

By delivering enhanced sensory input, MSS has potential to elevate brain arousal levels and optimize overall cognitive function, with benefits to language skills [17]. When patients are exposed to light and sound pulses at 40 Hz, the stimulation elicits brain rhythms in the gamma frequency range, which are critical for sustaining healthy brain activity [21]. A study conducted on audiovisual stimulation in older adults with MCI found that they exhibited significantly greater improvements in attentional control after training [22]. Video games incorporating multisensory integration have also been found to be effective in modulating prefrontal cortical function in older adults [23]. Furthermore, pleasurable multisensory experiences can improve emotional states and quality of life, which are critical factors for the efficacy of long-term cognitive interventions [24]. Existing MSS interventions most commonly involve combinations of visual, auditory, and tactile modalities, with audiovisual stimulation being the most frequently applied configuration, whereas multisensory approaches integrating 3 or more modalities (eg, visual-auditory-tactile) are widely implemented in Snoezelen-based environments [16,25].

However, current research findings are highly inconsistent, and no unified intervention protocols have been established. This heterogeneity primarily stems from several factors: first, the high diversity in study designs, such as variations in stimulus modality (eg, visual, auditory, tactile, and olfactory), intensity, duration, and frequency and total intervention length [25] and, second, the fact that few studies have explicitly related sensory stimulation parameters to cognitive outcomes, posing challenges in identifying the MSS components that drive effectiveness. Considering these challenges, it is essential to conduct a systematic review to synthesize current evidence to identify crucial aspects of interventions that most effectively improve cognitive function in older adults with MCI, thereby providing evidence-based guidance for future intervention designs. Therefore, the main research questions of this study are as follows: (1) what is the efficacy of MSS compared to control conditions on global and domain-specific cognitive function in older adults with MCI? (2) Which sensory modality combinations and intervention parameters are associated with greater cognitive improvements?

### Objectives

This systematic review aims to synthesize and evaluate existing scientific evidence on the effects of MSS on cognitive function in older patients with MCI. The specific objectives include (1) summarizing and critically appraising the efficacy of diverse multisensory interventions; (2) identifying the main limitations in the design and implementation of MSS interventions; and (3) providing guidance and recommendations for future high-quality, mechanism-oriented clinical trials targeting MSS for older adults with MCI.

## Methods

### Overview

This protocol adheres to the PRISMA-P (Preferred Reporting Items for Systematic Reviews and Meta-Analyses Protocols) checklist [26]. This systematic review also adheres to the recommendations of the PRISMA (Preferred Reporting Items for Systematic Reviews and Meta-Analyses) guidelines [27], strictly ensuring transparency in the research process and completeness of reporting. The protocol was registered in the PROSPERO platform (CRD420251161525).

### Eligibility Criteria

The literature selection will be guided by the following criteria structured according to the population, intervention, comparator, outcome, and study design framework.

#### Inclusion Criteria

Participants are adults aged 60 years or older with a confirmed diagnosis of MCI based on standardized neuropsychological assessments [26]. Interventions are MSS interventions involving at least 2 sensory modalities (eg, visual, auditory, tactile, olfactory, gustatory, or vestibular stimulation), with the aim of delivering MSS to influence cognitive outcomes. Stimuli should be presented simultaneously or in rapid succession within the same session so that they function as an integrated multisensory experience. Studies must report sufficient intervention detail, including the sensory modalities involved and the frequency and duration of stimulation. To enable consistent study selection and subgroup analyses, MSS interventions will be further classified into predefined operational categories, including but not limited to audiovisual MSS (eg, combined sound and light stimulation), visuotactile MSS (eg, interactive tactile tasks with visual feedback), auditory-tactile MSS (eg, vibration paired with sound cues), and multimodal combinations involving 3 or more senses (eg, visual-auditory-tactile or olfactory-auditory-visual). Delivery methods (eg, VR headsets, tablet-based systems, and physical environments) will be extracted and reported as contextual implementation characteristics and will be considered in subgroup analyses or meta-regression when sufficient data are available. To ensure transparent and rigorous synthesis, eligible comparator groups will be explicitly categorized into 2 distinct types: passive comparators (usual care or waitlist control), defined as participants receiving standard care or being placed on a waitlist without active treatment during the study period, and active comparators, defined as participants receiving an active non-MSS intervention (eg, single-sensory stimulation, cognitive training, or physical exercise). Studies must report at least one validated measure of cognitive function as an outcome, such as global cognitive function or performance in a specific cognitive domain (eg, memory, executive function, or attention). Eligible study designs are limited to RCTs published in English in peer-reviewed journals.

#### Exclusion Criteria

Studies will be excluded if (1) participants have a confirmed diagnosis of dementia or major neurological or psychiatric conditions that may confound cognitive assessment (eg, severe psychiatric disorders, traumatic brain injury, or end-stage renal or hepatic disease); (2) the interventions involve incidental sensory exposure or deliver different sensory modalities in separate sessions or with prolonged intervals; or (3) the study is a nonrandomized study, quasi-experimental study, observational study, qualitative study, conference abstract, dissertation, protocol, review, editorial, or other non–peer-reviewed publication.

### Information Sources

We will systematically search the following electronic databases to comprehensively identify relevant studies: PubMed, Embase, PsycInfo, Web of Science Core Collection, and the Cochrane Library. The database searches will span January 2011 to October 2025. The starting point of 2011 was selected because it marks the formal establishment of international standardized diagnostic criteria for MCI [28], ensuring the diagnostic homogeneity of the included participants; the end point of October 2025 was set to include the latest available evidence on MSS for older adults with MCI. Concurrently, we will screen the reference lists of all the included studies and relevant systematic reviews to identify additional eligible studies. For gray literature, we will search ProQuest Dissertations and Theses Global, OpenGrey, ClinicalTrials.gov, and the World Health Organization International Clinical Trials Registry Platform to identify unpublished or ongoing trials using the same core search terms as those used for the electronic databases. Corresponding authors will be contacted if core study data are missing from the retrieved articles

### Search Strategy

The search strategy was developed based on the key concepts of MCI, MSS, older adults, and cognitive outcomes. The search process and reporting will be in accordance with the PRISMA-S (Preferred Reporting Items for Systematic Reviews and Meta-Analyses literature search extension) guidelines [29] to enhance transparency, reproducibility, and completeness of the literature search. The full PubMed search strategy is presented in Table 1.

**Table 1.** Search strategy for PubMed.

Search number	Query
1	Multisensory stimulation[Title/Abstract] OR Multisensory exercise[Title/Abstract] OR Multisensory stimulation environment[Title/Abstract] OR Multisensory[Title/Abstract] OR Multisensory environmental therapy [Title/Abstract] OR Multisensory therapy[Title/Abstract] OR multisensory integration[Title/Abstract] OR sensory combination [Title/Abstract] OR audiovisual integration[Title/Abstract] OR Sensory stimulation[Title/Abstract] OR Snoezelen therapy[Title/Abstract] OR Snoezelen[Title/Abstract] OR Sensory integration[Title/Abstract] OR Visual stimulation[Title/Abstract] OR Olfactory stimulation[Title/Abstract] OR Acoustic Stimulation [Mesh] OR Acoustic stimulation[Title/Abstract] OR Auditory stimulation[Title/Abstract] OR Tactual stimulation[Title/Abstract]
2	Cognitive dysfunction[Mesh Terms] OR MCI [Title/Abstract] OR cognitive impairment[Title/Abstract] OR mild cognitive impairment[Title/Abstract] OR Cognitive impairment, mild[Title/Abstract] OR Cognitive dysfunction[Title/Abstract] OR Dysfunction, cognitive[Title/Abstract] OR Cognitive decline [Title/Abstract] OR Cognitive disorder[Title/Abstract] OR Decline, cognitive[Title/Abstract]
3	older adults[Title/Abstract] OR geriatric[Title/Abstract] OR elderly[Title/Abstract] OR Aged[Mesh]
4	cogniti*[Title/Abstract] OR cognition[Mesh Terms] OR executive function[Title/Abstract] OR executive function[Mesh Terms] OR memory[Title/Abstract] OR memory[Mesh Terms] OR orientation[Title/Abstract] OR orientation[Mesh Terms]
5	Randomized Controlled Trial [Publication Type] OR RCT [Title/Abstract] OR randomi* control*[Title/Abstract]
6	1 AND 2 AND 3 AND 4 AND 5

The PubMed search strategy was developed in consultation with a medical librarian and combines controlled vocabulary (MeSH [Medical Subject Headings] terms) with free-text keywords and synonyms to optimize sensitivity and specificity. Search terms within each concept will be combined using the Boolean operator “OR,” and blocks will be subsequently combined using “AND.” This strategy will be adapted for the other electronic databases by mapping MeSH terms to corresponding vocabulary and modifying free-text syntax as required by each database. For gray literature, simplified keyword-based search strategies derived from the same core concepts will be used to search ProQuest Dissertations and Theses Global and OpenGrey. ClinicalTrials.gov and the World Health Organization International Clinical Trials Registry Platform will be searched using the targeted condition (“mild cognitive impairment” OR “MCI”) and the intervention (“multisensory stimulation” OR “multisensory” OR “sensory stimulation”) to identify ongoing, completed, or unpublished studies. No date restrictions will be applied to gray literature searches to ensure comprehensive coverage. To enhance retrieval precision, we will apply a methodological filter for RCTs when searching database-specific controlled vocabularies allows it (eg, the Cochrane highly sensitive search strategy for RCTs in PubMed).

### Study Records

#### Data Management

All bibliographic records retrieved through database searches will be imported into EndNote (version 21.2; Clarivate Analytics), and duplicates will be removed. The selection will use Rayyan (Rayyan Systems Inc.), an internet-based tool for systematic reviews, data management, and data collection.

#### Selection Process

Initially, 2 reviewers (ZZ and WJ) will independently conduct a preliminary screening of the titles and abstracts to exclude irrelevant studies. Subsequently, the full texts of the remaining studies will be obtained and independently screened by 2 reviewers (SL and TX) according to the predetermined inclusion and exclusion criteria. Any discrepancies during screening will be resolved through discussion or consultation with a third reviewer (WG). The entire study selection process will be documented according to the PRISMA flow diagram, detailing the number of studies screened at each stage and the reasons for exclusion.

#### Data Collection Process and Data Items

A predesigned and pilot-tested data extraction form will be used to collect information. Extracted content will primarily include (1) study information (authors, publication year, country, study design, and sample size), (2) participant characteristics (MCI diagnostic criteria, age, gender, race, ethnicity, and baseline cognitive level), (3) intervention and control details (specific form of MSS [sensory modality and technology platform]; intervention duration, frequency, and intensity; and type of control group), (4) outcome measure data (cognitive measurements, mean values, SDs, and other statistics for each outcome measure at baseline and the postintervention time point), and (5) methodological quality information (eg, random sequence generation, allocation concealment, and blinding implementation).

Data collection will be performed independently by 2 reviewers and cross-checked to ensure accuracy.

### Outcomes and Prioritization

The primary outcome is improvement in cognitive function, including changes in global and domain-specific cognitive abilities such as memory, executive function, and attention, as measured using neuropsychological assessments. For global cognitive function, we will prioritize commonly used instruments such as the Mini-Mental State Examination (MMSE), Montreal Cognitive Assessment (MoCA), or Alzheimer’s Disease Assessment Scale–Cognitive Subscale. The secondary outcome is quality of life, such as physical, psychological, social, and emotional aspects.

If a study reports multiple measures for the same cognitive outcome, we will prioritize the measure most frequently used across the included studies to facilitate comparability. For studies reporting outcomes at multiple time points, postintervention assessments will be used for the primary analysis, with follow-up data extracted separately for exploratory analyses of sustainability.

To avoid double counting, when a single study reports multiple cognitive outcomes, we will apply a domain-based selection hierarchy. Specifically, for global cognition, the most comprehensive measure (MoCA or MMSE) will be selected. If a study uses multiple scales to assess cognitive function, we will use the MoCA scale, as one systematic review demonstrated that over 80% of the included articles indicated MoCA is superior to the MMSE in discriminating between individuals with MCI and those with no cognitive impairment. The mean area under the curve value for the MoCA was significantly larger than that of the MMSE in discriminating between participants with MCI and controls (0.883, 95% CI 0.855-0.912 vs 0.780, 95% CI 0.740-0.820 for the MMSE; *P*<.001) [30]. For domain-specific outcomes, we will select one primary measure per cognitive domain (eg, memory, executive function, and attention) based on the following hierarchy: (1) the study’s prespecified primary outcome, (2) the measure most frequently used across the included studies for that domain, or (3) a composite or average effect size calculated for that domain when no single measure meets the aforementioned criteria. If a study contributes multiple effect sizes from the same domain without a clear hierarchy, we will use a within-study pooled estimate to avoid unit-of-analysis error.

Given that implementation is a stated objective of this review, we will also extract and synthesize implementation-related outcomes, including adherence and safety. Adherence will be assessed based on indicators such as attendance rates, dropout rates, session completion rates, and reasons for withdrawal or noncompletion as reported in the included studies. Safety outcomes will include any reported adverse events or unintended effects associated with the MSS interventions regardless of whether they are classified as serious or nonserious. These implementation outcomes will be summarized narratively to provide insights into the feasibility, acceptability, and potential risks of MSS interventions in real-world settings.

### Risk of Bias

The risk of bias will be assessed using version 2 of the Cochrane risk-of-bias tool for randomized trials [31]. This tool will be applied to evaluate 5 major domains: bias from the randomization process, bias due to deviations from the intended interventions, bias due to missing outcome data, bias in the measurement of the outcome, and bias in the selection of the reported results. The risk of bias will be independently assessed by 2 reviewers (LQ and XW) through in-person discussions or consultation with a third reviewer (WG) to resolve discrepancies. The results will be presented as risk-of-bias graphs and summary tables.

### Data Synthesis

A narrative synthesis will be performed to summarize and present the main characteristics of the included studies in tables, including the MSS design, participant characteristics, intervention details, outcome measures, and main results. Additionally, we will describe the implementation-related factors reported across the studies, which may be specific to a particular study or health-related outcome. These include participant feedback, details of the intervention process, specific program components, and various contextual factors influencing implementation.

If 2 or more studies are sufficiently comparable in terms of sensory components, population characteristics, study and intervention design, and outcome measures, the study data will be quantitatively synthesized via meta-analysis using the Review Manager software (version 5.4; The Cochrane Collaboration) or R (*meta* package; R Foundation for Statistical Computing). Otherwise, a narrative synthesis will be conducted. For continuous outcomes, postintervention scores will be preferred over change scores when both are available as they are more commonly reported and allow for more straightforward interpretation. However, if change scores are reported more consistently across the included studies, change scores will be used for synthesis, and this will be clearly documented in the analysis. We will calculate the mean difference with a 95% CI for consistent cognitive function measurement tools to reflect changes after the intervention for the primary outcome of improvement in cognitive function. The standardized mean difference will be calculated if the studies used different tools.

Given the anticipated clinical and methodological diversity across studies, the meta-analyses will be performed using a random-effects model. This choice is made on the assumption that the true effect of MSS is likely to vary across populations and intervention contexts. Statistical heterogeneity will be described using the *I*^2^ statistic, and if sufficient studies are available, 95% prediction intervals will also be calculated for pooled random-effects estimates [32] to support interpretation of the pooled effects. The interpretation of heterogeneity will also consider the magnitude and direction of effect estimates as well as the clinical and methodological characteristics of the included studies. For random-effects meta-analyses, we will use the Hartung-Knapp-Sidik-Jonkman method to estimate the pooled effect and its CI rather than the conventional DerSimonian-Laird method. The Hartung-Knapp-Sidik-Jonkman method has been shown to provide more adequate error rates and greater precision, particularly when the number of studies is small or when between-study heterogeneity is present [33].

A meta-regression will be performed to analyze the differences in cognitive improvement among the different MSS designs. Subgroup analyses or meta-regression will be considered when at least 10 studies are available for inclusion, as recommended by the *Cochrane Handbook for Systematic Reviews of Interventions* [34]. Potential subgroup variables include (1) type of MSS, (2) sensory modality combinations (eg, audiovisual vs visuotactile), (3) intervention duration (eg, ≤8 weeks vs >8 weeks), (4) session frequency, and (5) MCI subtype (eg, amnestic vs nonamnestic MCI) if data permit. A sensitivity analysis will be conducted by excluding studies with large or extreme effect sizes. Where sufficient data are available, separate meta-analyses will be conducted for each comparator category. For studies including more than one eligible comparator group, the passive comparators will be prioritized for the primary analysis because they provide a more consistent and clinically interpretable baseline, allowing for the estimation of the absolute effect of MSS while minimizing the heterogeneity introduced by diverse active control conditions.

Funnel plots and the Egger test will be used to examine small-study effects. The funnel plot asymmetry or statistically significant Egger test results will be interpreted cautiously as these may reflect a range of factors, including publication bias, methodological qualities, true heterogeneity, or chance [35,36].

### Confidence in Cumulative Evidence

The quality of the evidence will be assessed using the Grading of Recommendations Assessment, Development, and Evaluation (GRADE) framework [37]. Notably, the GRADE framework provides a structured approach for evaluating and grading evidence quality, which is categorized into the following levels: high, moderate, low, and very low. The quality of the evidence will be independently assessed by 2 reviewers (LQ and XW). Discrepancies will be resolved through face-to-face discussions or consultations with a third reviewer (WG). The GRADE evaluation results will be presented in summary tables.

## Results

This systematic review is currently in the protocol design stage, with all research procedures and methodological frameworks formulated and confirmed. The systematic literature search was conducted from November 1, 2025, to December 31, 2025, and a total of 956 related studies were retrieved; the expected publication date of the relevant results is October 2026. Study selection and double-independent data extraction were conducted from January 1, 2026, to February 28, 2026; and data synthesis, statistical analysis, and manuscript preparation are expected to be completed by April 30, 2026.

## Discussion

### Anticipated Principal Findings

The systematic review is expected to provide a comprehensive synthesis of the available evidence on the effectiveness of MSS in improving cognitive function or delaying cognitive decline among older adults with MCI. The completed review is also anticipated to identify which sensory combinations and patterns of stimulus synchrony may be associated with greater cognitive benefits. Additionally, the review will provide an overview of limitations in intervention design, feasibility, and adherence across existing studies, with the goal of contributing to the development of more effective and evidence-based intervention strategies for older adults with MCI.

### Comparison to Prior Work and Contributions of the Review

As an NPI, MSS has shown potential benefits in cognitive intervention research. However, existing studies have primarily focused on patients with dementia or Alzheimer disease [38]. A study used the combination of sound and light stimulation as a noninvasive approach to modulate neuron activity and has suggested that it could induce the brain lymphatic function and promote the clearance of β-amyloid from the neurons’ microenvironment [39]. Given its noninvasiveness and practical feasibility, MSS represents a promising intervention method. Nevertheless, whether MSS produces differential effects across stages of cognitive impairment remains unclear and needs further exploration.

Another important issue is that limited attention has been paid to how different sensory combinations may influence intervention outcomes. A recent systematic review of the effect of VR-based interventions on cognitive outcomes in older adults with subjective cognitive decline and older adults with MCI found that VR-based therapies were associated with improvements in both subjective cognitive concerns and objective cognitive performance [40]. Although this finding supports the potential value of MSS, the intervention protocols in these studies did not sufficiently describe or compare the specific forms and parameters of visual, auditory, and other sensory stimuli. Therefore, the underlying mechanisms remain insufficiently understood, particularly from the neurophysiological perspective. At the same time, existing studies have shown that multisensory approaches may be beneficial in rehabilitation and may induce neuroplastic changes within cortical networks, suggesting potential advantages of MSS [41]. Against this background, the planned review is expected to provide a more detailed synthesis of MSS modalities, sensory combinations, and effectiveness and may help inform the design of future interventions and related research.

### Limitations

Although the development of this systematic review will adhere to rigorous methodological standards, several limitations should be acknowledged. First, given the emerging nature of MSS research in older adults with MCI, the included studies may exhibit heterogeneity in sample sizes, intervention protocols, and outcome measures, which could affect the comparability of the findings. To address this, we will use random-effects models, and where data permit, we will conduct subgroup analyses and meta-regression to explore sources of heterogeneity. Second, the definition and reporting quality of MSS interventions may vary considerably across studies. We will address this by systematically extracting intervention details using the Template for Intervention Description and Replication (TIDieR) checklist [42] and explicitly evaluating the impact of reporting adequacy on the overall evidence.

### Future Directions

The findings of the completed systematic review may help guide the next stage of research on MSS for older adults with MCI. Future studies may need to move beyond evaluating overall effectiveness and place greater emphasis on identifying the active components of MSS interventions. There is also a need for more robust trials with improved methodological quality, standardized cognitive outcome measures, and adequate follow-up to assess the long-term intervention effects. Moreover, research integrating behavioral outcomes with neurophysiological or neuroimaging measures may help clarify the mechanisms through which MSS exerts its effects and support the development of more targeted interventions.

### Dissemination Plan

Once the systematic review is completed, the findings will be disseminated through publication in a peer-reviewed journal and presentation at academic conferences. The results will also be shared with researchers, clinicians, and other stakeholders involved in cognitive health and aging research to support evidence-based practice and future intervention development.

### Conclusions

This protocol describes the design of a systematic review to evaluate the effectiveness of MSS on cognitive function in older adults with MCI. The planned review is expected to provide a synthesis of the available evidence, summarize intervention characteristics, and identify limitations in the existing literature. The findings will help inform future research and the development of NPIs to support cognitive health in older adults with MCI.

## Supplementary material

10.2196/88720 Multimedia Appendix 1 Artificial intelligence use disclosure.

10.2196/88720 Checklist 1 PRISMA-P checklist.

## Abbreviations

DCT: digital cognitive training
GRADE: Grading of Recommendations Assessment, Development, and Evaluation
MCI: mild cognitive impairment
MeSH: Medical Subject Headings
MMSE: Mini-Mental State Examination
MoCA: Montreal Cognitive Assessment
MSS: multisensory stimulation
NPI: nonpharmacological intervention
PRISMA: Preferred Reporting Items for Systematic Reviews and Meta-Analyses
PRISMA-P: Preferred Reporting Items for Systematic Reviews and Meta-Analyses Protocols
PRISMA-S: Preferred Reporting Items for Systematic Reviews and Meta-Analyses literature search extension
RCT: randomized controlled trial
TIDieR: Template for Intervention Description and Replication
VR: virtual reality
