# Supplementary material for: Effectiveness of Multisensory Stimulation on Cognitive Function in Older Adults With Mild Cognitive Impairment: Protocol for a Systematic Review
Source: JMIR Res Protoc. 2026 May 14;15:e88720. doi: 10.2196/88720 (PMC13175526; doi:10.2196/88720)
Supplement: Multimedia Appendix 1 [file resprot-v15-e88720-s001.docx]

Appendix 1

Disclosure of Delegation to Generative AI

The authors declare the use of generative AI in the research and writing process. According to the GAIDeT taxonomy (2025), the following tasks were delegated to GAI tools under full human supervision:

- Proofreading and editing

- Adapting and adjusting emotional tone

- Translation

The GAI tool used was: DeepL and ChatGPT-4.0.

Responsibility for the final manuscript lies entirely with the authors.

GAI tools are not listed as authors and do not bear responsibility for the final outcomes.

Declaration submitted by: Collective responsibility

Additional note: We used AI tools to assist with English proofreading and translation, as the authors are non-native English speakers.
